# A Possible Case of Autoimmune Encephalitis After mRNA COVID-19 Booster Vaccine: A Case Report

**DOI:** 10.7759/cureus.31118

**Published:** 2022-11-05

**Authors:** Mohammad Abu-Abaa, Ghassan Dawood, Hassaan Arshad, Omar Jumaah, Daniel Landau

**Affiliations:** 1 Internal Medicine Residency Program, Capital Health Regional Medical Center, Trenton, USA; 2 Neurology, Capital Health Regional Medical Center, Trenton, USA

**Keywords:** stroke-like symptoms, behavioral changes, global aphasia, acute encephalitis, covid-19 vaccine

## Abstract

As the use of COVID-19 vaccines gains more prevalence, rare and uncommon side effects are reported in the medical literature. This is a case report of a 75-year-old male patient who presented on the second day after receiving the Moderna Bivalent mRNA COVID-19 booster vaccine with abrupt onset behavioral changes and global aphasia with no focal deficits. Stroke and infectious meningitis/encephalitis were ruled out. Signs of aseptic inflammation were seen on cerebrospinal fluid (CSF) analysis. Workup for autoimmune and paraneoplastic encephalitis was unyielding. The observation of rapid clinical improvement prompted watchful waiting that concluded in the resolution of clinical manifestations within less than a week of onset. This case is reported to support the currently limited knowledge of rare neurological sequelae of mRNA vaccine and is in line with recently published few cases that suggest vaccine-related encephalitis.

## Introduction

Rare neurological sequelae have been more reported in association with ChAdOx1nCoV-19 vaccines (Oxford-AstraZeneca). However, serious neurological outcomes of mRNA COVID-19 vaccination have been less well reported in the literature [1]. Previously, vaccine-induced encephalitis has already been described in vaccination for influenza, hepatitis B, Haemophilus influenzae type B, measles-mumps-rubella, tetanus, diphtheria, pertussis, polio, and Japanese encephalitis [2]. Several neurological sequelae in association with the COVID-19 vaccine were reported including Guillain-Barré syndrome (GBS), transverse myelitis, acute disseminated encephalomyelitis (ADEM), stroke, cerebral venous sinus thrombosis (CVST) and cranial neuropathies [3]. Multiple mechanisms of vaccine-induced encephalitis were suggested and are reviewed here. This case shows a possible autoimmune encephalitis that was likely contributed to by the mRNA COVID-19 vaccine.

## Case presentation

**Figure 1:**
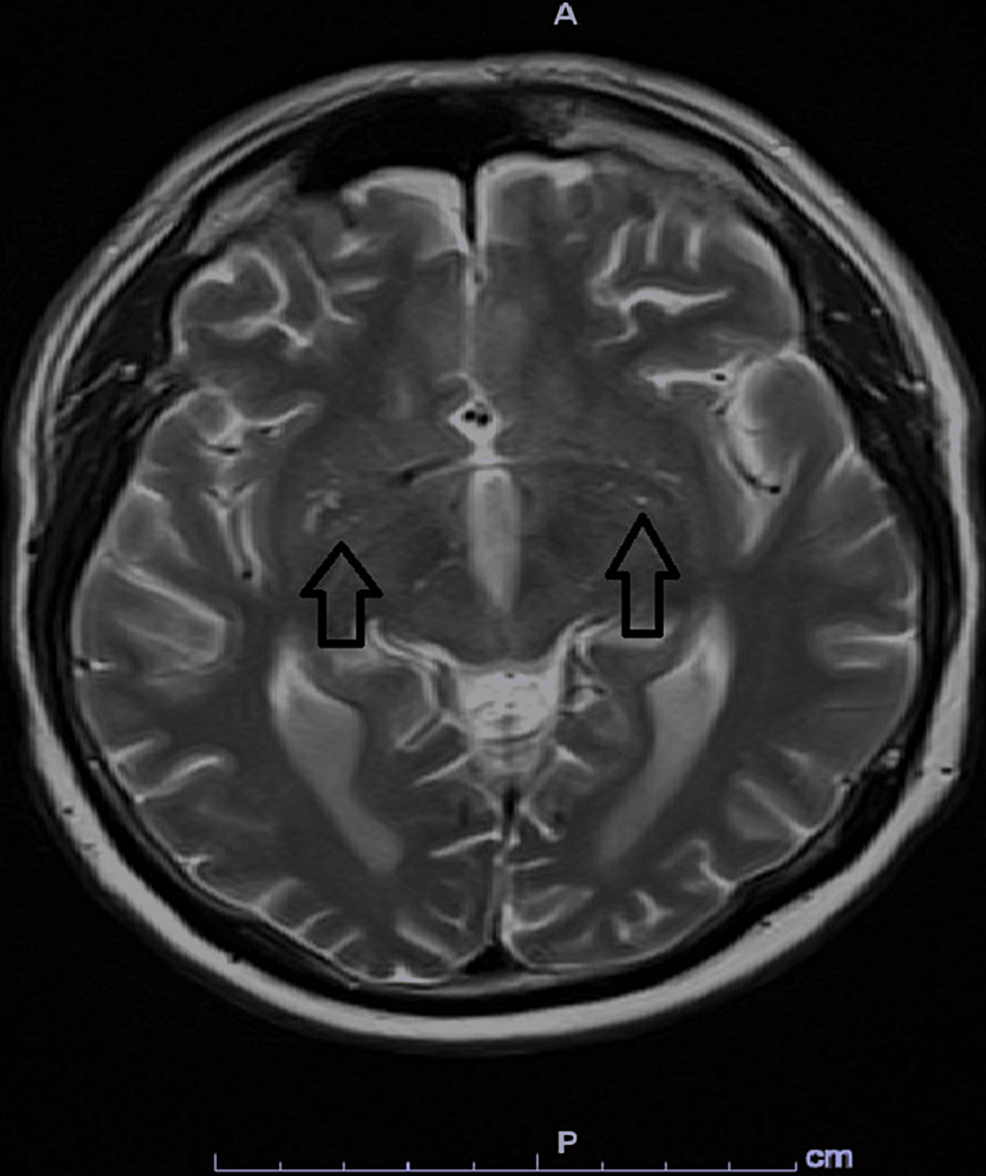
A T2 MRI with no evidence of lesion nor leptomeningeal enhancement.

A 75-year-old male patient presented with several hours of history of altered mental status, aphasia, and behavioral changes. It was reported that he received a booster dose of Moderna Bivalent mRNA COVID-19 vaccine one day prior to presentation. On the next day, he had abrupt behavioral changes where he started to attempt eating a disposable plastic plate, having poor attention, urinating in a public place and abrupt onset of aphasia. His past medical history was remarkable only for coronary artery disease, hypertension, and diabetes. No prior COVID-19 infection was reported. He had received three doses of the Pfizer-BioNTech COVID-19 vaccine. No prior record was available regarding his prior vaccination history. In the emergency department (ED), he was afebrile and his vital signs were stable. Physical exam showed global aphasia and bilateral Babinski sign with no focal neurological sign. Nuchal rigidity as well as other signs of meningeal irritation was also negative including Kerning’s sign and Brudziniski's sign. Basic lab workup including complete blood count, comprehensive metabolic panel, and drug screen was also unremarkable. Acute stroke was ruled out by computed tomography (CT) head, CT angiography (CTA) head and neck, and also magnetic resonance imaging (MRI) that showed only periventricular hyperintensities with no diffusion restriction suggestive of chronic microvascular disease and mild cerebral atrophy. No meningeal enhancement was seen (Figure 1). 

**Figure 2:**
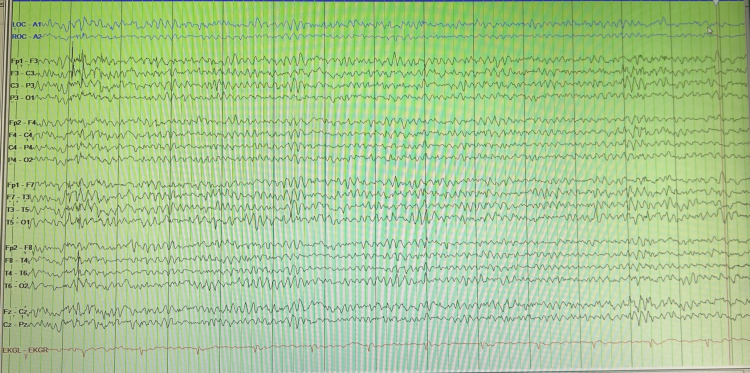
EEG findings are nonspecific and include posterior dominant rhythm of mixed theta and delta waves with superimposed faster frontotemporal delta waves but with no epileptiform discharges or focal slowing.

Inflammatory markers were not elevated. Cerebrospinal fluid (CSF) analysis showed lymphocytic pleocytosis with a slight elevation of protein as shown in Table 1. The CSF was negative for cryptococcal, Epstein Barr virus (EBV), cytomegalovirus (CMV), and venereal disease research lab (VDRL) test. The CSF acid-fast staining was negative even after one week of incubation. Meningitis/encephalitis panel including Cryptococcus, varicella-zoster virus (VZV), Streptococcus, Neisseria, Listeria, human Parechovirus, human herpes virus 6, human simplex virus type 1 and 2, H. influenzae, Escherichia coli K1, Enterovirus, and Cytomegalovirus were also negative. A paraneoplastic panel including anti-Hu, anti-Yo, and anti-Ri antibodies was negative. Anti-NMDR and anti-Ma2 antibodies were also nonreactive. Two pairs of oligoclonal bands were observed in CSF and serum. Myelin basic protein was elevated at 29.6 ng/mL (reference 0-5.6 ng/mL). Blood culture as well as urinalysis were unyielding. Meningitis/encephalitis panel including West Nile Virus (WNV) and Lyme serology was non-reactive. A 72-hour electroencephalogram (EEG) showed posterior dominant rhythm of mixed theta and delta waves with superimposed faster frontotemporal delta waves but with no epileptiform discharges or focal slowing (Figure 2). No active therapeutic measure was given, and the patient was watchfully monitored as signs of rapid improvement were appreciated. Physical exam findings during hospitalization showed gradual improvement over the five days course of hospitalization. Aphasia resolved and no further behavioral changes were seen. At the end of hospitalization, he was neurologically intact and discharged home.

**Table 1:** CSF analysis showing lymphocytic pleocytosis with slightly elevated protein. Picture is compatible with aseptic encephalitis.

CSF Variable	Finding	Reference Range
White Blood Cell Count	9 cells/mL	Less than 5 cells/mL
Lymphocyte Percentage	62%	40%-80%
Protein	76 mg/dL	12-60 mg/dL
Glucose	75 mg/dL	40-70 mg/dL

## Discussion

The safety profile of these vaccines needs continuous monitoring. Hence, post-marketing pharmacovigilance remains of vital importance to establish an accurate safety profile of COVID-19 vaccines [4]. Our patient meets the diagnostic criteria of possible autoimmune encephalitis including rapid onset of behavioral changes and altered mental status, CSF pleocytosis, and reasonable exclusion of other etiologies [5]. No evidence of infectious etiology was seen on the meningitis/encephalitis panel. Also, the paraneoplastic panel was negative. Although it is difficult to establish a cause-effect relationship between the vaccine and acute encephalitis, the basis of our suggestion is the close temporal relationship between the onset of encephalitis and administration of the COVID-19 vaccine, abrupt onset of symptoms, relative rapid improvement without therapeutic intervention as well as lack of brain lesions on imaging.

Only a few cases describing a possible COVID-19 vaccine-related encephalitis have been reported in English literature [6,7]. Three cases of Oxford-Astrazeneca vaccine-related autoimmune encephalitis were reported. One presented with opsoclonus-myoclonus syndrome and two presented with seizure, gait disturbance, and cognitive decline [8]. Three cases of COVID-19 vaccine (Moderna) related encephalopathy with no MRI findings were also reported [9,10]. Those patients had psychiatric symptoms and were believed to have had cytokine storms. No such finding of cytokine storm was seen in the above-mentioned case. Also, the onset of symptoms in our patient was earlier as compared to the other cases. The general belief is still that the benefits of the COVID-19 vaccine outweigh the potential risks.

The neurotropism of the COVID-19 virus has been well documented. Neurological complications of COVID-19 can include anosmia, acute transverse myelitis, acute demyelinating encephalomyelitis, neuromyelitis optica, and acute motor axonal neuropathy [11]. The mechanism is likely either direct viral neurotoxicity or neuronal damage by the host inflammatory response. It is not established yet that the COVID-19 vaccine can induce such neuronal toxicity. However, it is possible to hypothesize that molecular mimicry or nonspecific immune bystander activation is responsible for these vaccine-related complications [12,13]. Regarding association with CNS demyelinating disorders reported in association with the COVID-19 vaccine, the vaccine may act as a trigger to demyelinating disease in those with subclinical disease or with genetic disposition. However, no causality has been established yet [14]. Also, the mRNA-based vaccine may lead to spike protein expression which may contribute to inflammatory complications [15]. While it was not seen in our patient, recent studies also indicate that the mRNA vaccine in patients with prior COVID-19 infection can elicit a stronger humoral and cellular response and induce neurological damage [16].

According to Vaccine Adverse Events Reporting System (VAERS) and Medicine and Healthcare products Regulatory Agency (MHRA), the most commonly reported neurological side effect of the COVID-19 vaccine is ischemic events [17,18]. A recent systematic review of 30 reported CNS neurological sequelae of the COVID-19 vaccine concluded that the most common is CVST [3]. This was seen mostly in association with the Oxford-AstraZeneca vaccine in 93% and with the Moderna vaccine in 7% of cases. However, this finding may reflect the fact Oxford-AstraZeneca is the most commonly approved vaccine. The same review reported three cases of vaccine-induced encephalitis/encephalopathy, two presenting with status epilepticus and one with autoimmune encephalitis. Unlike our patient, these reactions were observed after the first dose of the Moderna vaccine.

## Conclusions

Autoimmune encephalitis secondary to the COVID-19 vaccine has been less reported in the literature in association with the mRNA vaccine. It may or may not be associated with cytokine storms. It may present with psychotic symptoms, seizure, gait disturbance, and/or neurological deficits. Association is based on temporal relationship and true causality is difficult to establish. Although immunosuppression with steroid or intravenous immunoglobulin has been pursued in the reported cases of COVID-19 vaccine-associated encephalitis, watchful waiting in our patient with no evidence of cytokine storm concluded in a rapid spontaneous recovery.
